# Allostatic Load and its Impact on amyloid, tau, and hippocampus: a potential underlying mechanism explaining variability in Alzheimer's Disease pathology and progression

**DOI:** 10.1002/alz.091155

**Published:** 2025-01-09

**Authors:** Juliana Nery Souza‐Talarico, Maria Hein, Yelena Perkhounkova, Jihye Lee

**Affiliations:** ^1^ University of Iowa, Iowa, IA USA; ^2^ University of Iowa, Iowa City, IA USA; ^3^ University of Iowa College of Nursing, Iowa City, IA USA

## Abstract

**Background:**

Accumulation of β‐amyloid (Aβ) and tau proteins can predict the risk of Alzheimer's disease (AD) at asymptomatic stages and are promising measures for screening individuals at risk. However, not all individuals with Aβ and tau pathology progress to AD; some remain cognitively healthy. That variability challenges prediction accuracy and incorporation of AD pathology into clinical practice. Identifying underlying mechanisms that accelerate AD progression is critical to understanding disease trajectories, assessing risk, and promoting timely intervention. Allostatic load (AL), or the "wear and tear" of chronic stress, has been linked to AD risk. However, whether AL influences AD pathology requires further investigation. We aimed to determine the relationship between AL, Aβ, tau, and hippocampus volume (HV).

**Methods:**

Using the Alzheimer's Disease Neuroimaging Initiative (ADNI) database, we analyzed longitudinal data from 385 older adults. Blood neuroendocrine (cortisol, LH, prolactin, testosterone) and immunologic (C‐reactive protein, fibrinogen, IL‐13, IL‐16, IL‐18, IL‐3, IL‐6, MCP‐3, MCP4, TNF‐alpha, insulin‐like growth factor) stress‐related biomarkers obtained at baseline and 12 months were combined into an AL index. Aβ42 measured in the blood (Aβ42_blood_) and cerebrospinal fluid (Aβ42_CSF_), CSF p‐tau proteins, and HV from brain MRI were analyzed over 24 months. Linear mixed models were adjusted for socioeconomic and APOE4 covariates.

**Results:**

Higher AL increases over 12 months were associated with higher elevation on p‐tau (p=.007; 95%CI [‐.927,‐.147]), and Aβ42_blood_ (p=.01; 95%CI [.282, 2.54]), and a trend to lower Aβ42_CSF_ (p=.07;95%CI [‐.032, .001]) over 24 months. An interaction between AL increase and APOE4 was observed, indicating that higher increases in AL were associated with higher Aβ42_blood_ but for APOE4 with two E4 copies only (p=.03;95%CI [‐2.568, ‐0.0908]). No association was observed with HV (p=.435).

**Conclusion:**

Amyloid and tau deposition over time was higher among participants with increases in AL, suggesting that elevated AL profiles might signal a potential underlying mechanism explaining AD pathology trajectories. Further research is needed to examine whether incorporating AL in AD models improves the prediction of cognitive decline. A critical step for non‐pharmacological clinical trials testing interventions to reduce dementia risk.

